# Neuropsychiatric Systemic Lupus Erythematosus Diagnosed Following Treatment Initiated for Acute Encephalitis

**DOI:** 10.7759/cureus.102396

**Published:** 2026-01-27

**Authors:** Keidai Kumazawa, Norio Nakagawa, Koichi Tanda, Yasuko Okumura, Akira Nishimura

**Affiliations:** 1 Department of Pediatrics, Kyoto Prefectural University of Medicine, Kyoto, JPN; 2 Department of Pediatrics, Japanese Red Cross Kyoto Daiichi Hospital, Kyoto, JPN

**Keywords:** acute encephalitis, neuropsychiatric systemic lupus erythematosus, pediatric, renal dysfunction, seizure

## Abstract

Systemic lupus erythematosus (SLE) is a systemic autoimmune disease characterized by diverse clinical manifestations. This case report describes a 14-year-old female patient diagnosed with neuropsychiatric SLE (NP-SLE) following status epilepticus. The patient was a 14-year-old previously healthy female and was transported to the emergency department owing to fever and status epilepticus. Based on imaging findings, clinical findings, and the patient's age, anti-N-methyl-D-aspartate (NMDA) receptor encephalitis (NMDARE) was suspected, and intensive care was initiated. However, persistent renal dysfunction and cytopenia prompted detailed investigation, leading to a diagnosis of NP-SLE. SLE is a systemic disease requiring long-term treatment. In such cases, where characteristic rashes are absent, differential diagnosis based on physical findings is difficult. Although NP-SLE and NMDARE share many standard features, including clinical symptoms and age of onset, measuring antinuclear antibody (ANA) and complement levels may be valuable in the differential diagnosis.

## Introduction

Systemic lupus erythematosus (SLE) is a prototypic systemic autoimmune disease characterized by loss of immune tolerance, production of a wide array of autoantibodies, immune-complex deposition with complement activation, and dysregulation of innate and adaptive immune responses, leading to diverse clinical manifestations [1]. In childhood-onset SLE, lupus nephritis and neuropsychiatric SLE (NP-SLE), manifesting with psychiatric and neurological symptoms, have a significant impact on prognosis [2]; however, diagnosis of the latter is often challenging because the symptoms are diverse and affect multiple organs [3]. We report the case of a 14-year-old girl who presented with seizures, was initially diagnosed with anti-N-methyl-D-aspartate (NMDA) receptor encephalitis (NMDARE) based on clinical and imaging findings, and began treatment accordingly, but was later diagnosed with NP-SLE.

## Case presentation

A 14-year-old girl with no prior medical history and a four-day history of fever was taken to the emergency room with clonic seizures lasting >30 min. Her physical examination findings were as follows: body temperature, 38.1ºC; blood pressure, 78/39 mmHg; heart rate, 100 beats per min; respiratory rate, 16 breaths per min; and SpO2, 99% under 10 L oxygen. Physical examination revealed no significant findings in heart sounds or breath sounds, and no edema or rash was observed. Her level of consciousness was Glasgow Coma Scale-E1V1M1 (Japan Coma Score: 300) and impaired. A peripheral venous line was secured, and midazolam (0.16 mg/kg) was administered intravenously to achieve sedation. Intubation was performed to stabilize respiratory and circulatory function while initiating fluid resuscitation, and mechanical ventilation was initiated.

Her blood test results showed elevated creatinine levels (132.6 µmol/L, reference value: 40.7-62.8 µmol/L) and a decreased platelet count (88000/µL) (Table 1). No decrease in white blood cell count (10680/µL) or lymphocyte count (4160/µL) was observed. Systemic management was initiated in the intensive care unit. In addition to continuous administration of midazolam, dexmedetomidine, and fentanyl, thiopental was administered to achieve deep sedation. Furthermore, brain temperature stabilization therapy was implemented, and mannitol and edaravone were administered for brain protection.

**Table 1:** Various test results NMDA: N-methyl-D-aspartate; HPF: high-power field

Blood tests on admission				Blood tests (day 4)				Spinal fluid tests (day 1)		
Parameters	Value	Reference value		Parameters	Value	Reference value		Parameters	Value	Reference value
White blood cell (/µL)	10680	3500-9000		Complement c3 (g/L)	0.31	0.69-1.28		Cell count (×10⁶/L)	5	0-5
Lymphocyte (/µL)	4160	1000-4800		Complement c4 (g/L)	0.01	0.11-0.34		Protein (mg/L)	4480	100-400
Red blood cell (×10⁶/µL)	3.93	3.80-5.04		Hemolytic complement activity (U/mL)	＜3	31.6-57.6		Glucose (mmol/L)	5.7	2.5-4.4
Hemoglobin (g/L)	115	116-148		Anti-Sm antibody	Negative			Interleukin-6 (pg/mL)	376.8	＜7
Platelets (×10⁴/µL)	8.8	15.8-34.8		Anti-ribosomal P antibody (U)	＜6	＜6		Film array	Negative	
Total protein (g/L)	64	66-81						Culture test	Negative	
Albumin (g/L)	32	41-51		Venous blood gas analysis on admission						
Aspartate aminotransferase (U/L)	47	13-30		Parameters	Value	Reference value		Spinal fluid tests (day 6)		
Alanine aminotransferase (U/L)	21	7-23		Potential hydrogen	6.756	7.35-7.45		Parameters	Value	Reference value
Lactate dehydrogenase (U/L)	480	124-222		Partial pressure of carbon dioxide (Torr)	104	35.0-45.0		Anti-NMDA receptor antibody	Negative	
Total bilirubin (µmol/L)	8.55	6.83-25.62		Hydrogen carbonate (mmol/L)	14.6	20.0-26.0		Myelin basic protein (ng/mL)	0.167	＜0.102
Direct bilirubin (µmol/L)	1.71	＜5.12		Base excess (mmol/L)	-21.7	-3.0-3.0		Oligoclonal bands	Negative	
Creatine phosphokinase (U/L)	295	41-153		Lactate (mmol/L)	1.45	0.5-1.5				
Amylase (U/L)	164	44-132								
Blood urea nitrogen (mmol/L)	9.6	2.9-7.1						Urinalysis findings (day 1)		
Creatinine (µmol/L)	132.6	40.7-62.8						Parameters	Value	Reference value
C-reactive protein (mg/L)	0.2	＜1.4						Red blood cell (/HPF)	＞100	3-4
Sodium (mmol/L)	134	138-145						Urine protein-to-creatinine ratio (g/gCr)	3.554	＜0.15
Ammonia (µmol/L)	282	7.0-38.8						White blood cell (/HPF)	1-4	3-4
Antinuclear antibodies	×320	＜×40								
Homogeneous	×80	＜×40						Urinalysis findings (day 22)		
Speckled	×320	＜×40						Parameters	Value	Reference value
Anti-ds-DNA antibodies (U/mL)	194	＜12.0						Red blood cell (/HPF)	30-49	3-4
								Urine protein-to-creatinine ratio (g/gCr)	3.883	＜0.15
								White blood cell (/HPF)	1-4	3-4

Cerebrospinal fluid (CSF) analysis findings revealed increased protein (4480 mg/L) and IL-6 (15.1 pmol/L) levels, but no increase in the CSF cell count. The CSF culture was negative, and the CSF film array also showed no significant findings. Additionally, as part of the evaluation for other infectious diseases, blood cultures were negative, and polymerase chain reaction (PCR) tests for influenza and COVID-19 were also negative.

**Figure 1:**
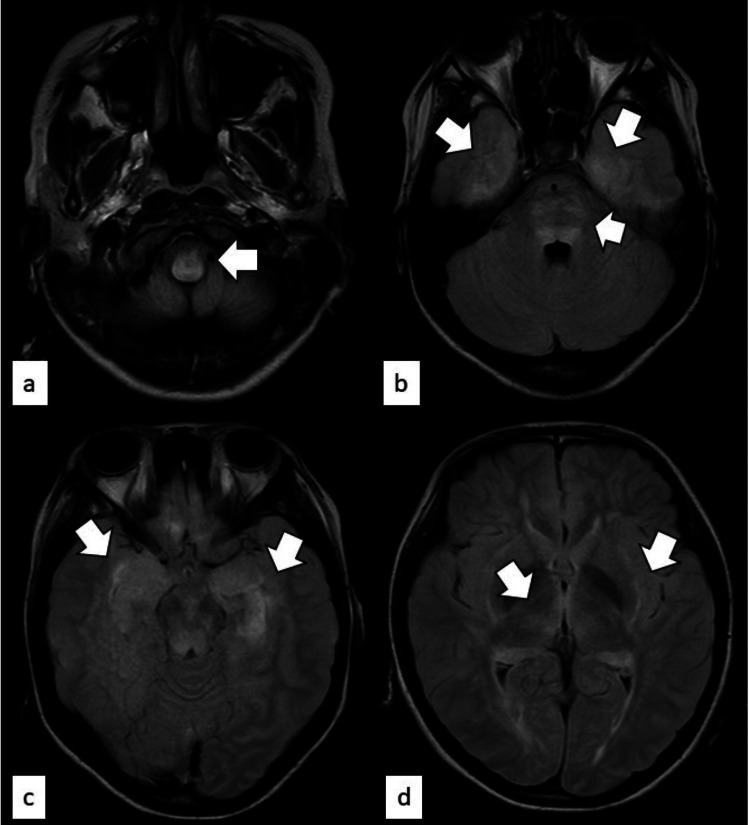
Magnetic resonance imaging scan (fluid attenuated inversion recovery) performed at the time of hospitalization High intensity regions are indicated by arrows as follows: (a) medulla oblongata, (b) medial temporal lobe and bridge capsule, (c) hippocampus, (d) thalamus and external capsule

Brain magnetic resonance imaging (MRI) showed bilateral symmetrical punctate high-signal areas in the medial temporal lobe, including the medulla oblongata, bridge capsule, dorsal midbrain, hippocampus, insular cortex, thalamus, and outer capsule (Figure 1). Awake electroencephalography showed high-voltage, irregular, slow waves with frontal sharp waves.

**Figure 2:**
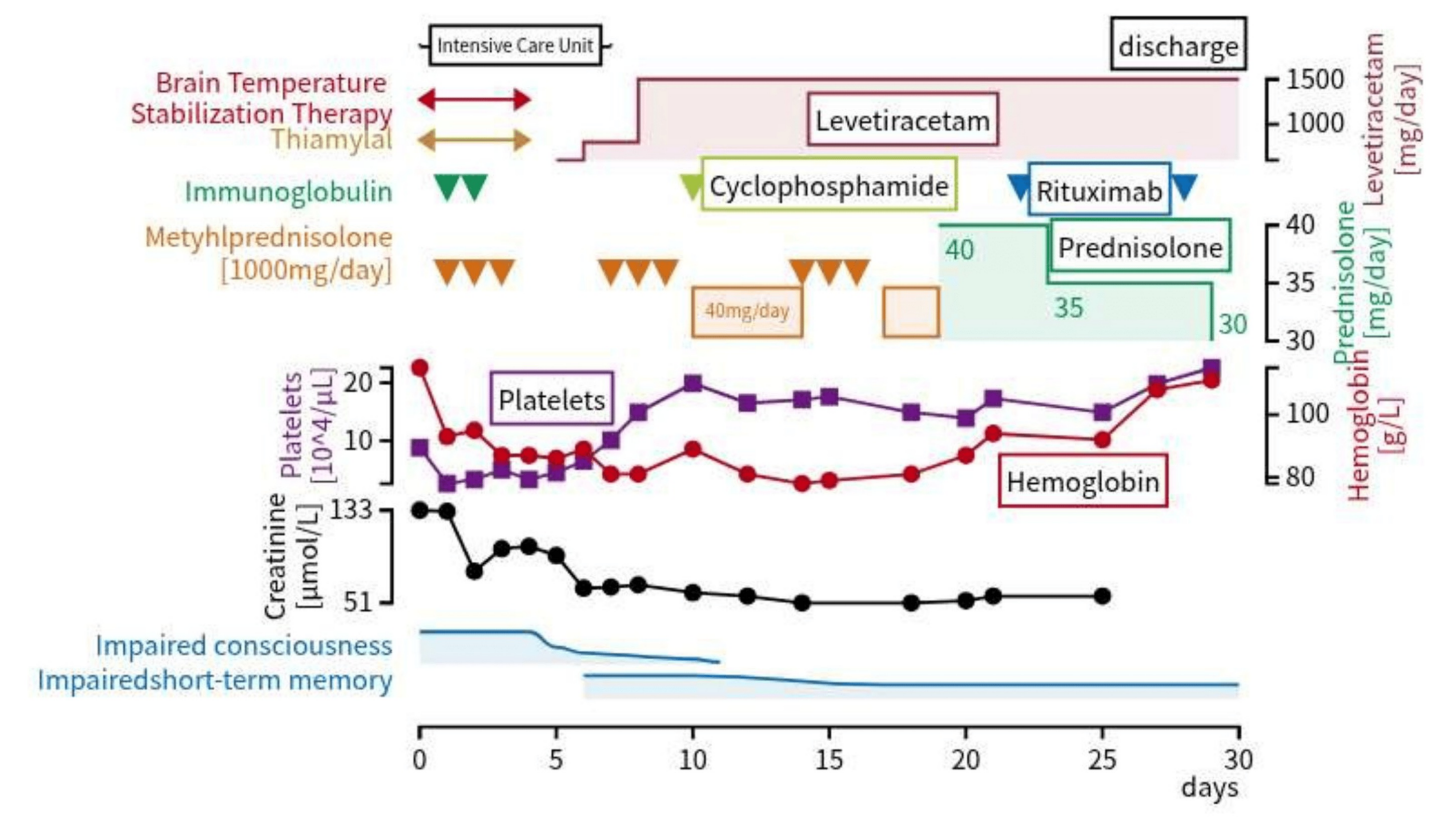
Course of treatment during hospitalization

The extensive bilateral symmetric lesions observed suggested the presence of inflammation throughout the brain, and the fact that the patient was a young woman made autoimmune encephalitis (especially anti-NMDA receptor antibody encephalitis) a likely cause. NMDARE was suspected, and the patient was treated with pulse intravenous (IV) methylprednisolone and high-dose IV immunoglobulin. The chest and abdominal CT scan did not reveal findings suggestive of ovarian teratoma, which may be associated with NMDARE. However, on hospital day 4, her blood test results were as follows: creatinine, 99.0 µmol/L; hemoglobin, 87 g/L; and platelets, 50000/µL, indicating persistent renal dysfunction, anemia, and thrombocytopenia. Additional tests revealed positive antinuclear antibodies (ANA) (×320; homogeneous, ×80; speckled, ×320), elevated anti-dsDNA antibodies (194 U/mL), and decreased complement levels (C3, 310 mg/L; C4, 10 mg/L; and hemolytic complement activity, ＜3 U/mL). She was diagnosed with NP-SLE based on the 2019 European League Against Rheumatism and American College of Rheumatology criteria. On hospital day 6, the CSF test was repeated, and she tested negative for anti-NMDA receptor antibodies (cell-based assay). To treat the NP-SLE, three courses of pulse IV methylprednisolone and one course of IV cyclophosphamide were administered. The Disease Activity in SLE (SLEDAI) score, used to assess disease activity in SLE, was 36 points on the seventh day of hospitalization (seizures, organic brain disorder, headache, hematuria, proteinuria, hypocomplementemia, elevated anti-dsDNA antibodies). Subsequently, her creatinine levels improved over time, but her anemia (hemoglobin: 88 g/L) and thrombocytopenia (platelets: 149,000/µL) persisted, and her short-term memory impairment persisted. The SLEDAI score was 10 points (hematuria, proteinuria, hypocomplementemia) owing to insufficient efficacy. Therefore, rituximab was started as an additional treatment on hospital day 22 (four courses were administered). A renal biopsy revealed mesangial proliferative glomerulonephritis. Hematuria and proteinuria were present at admission but did not worsen; they gradually improved. She was discharged from the hospital following improvement in her anemia and thrombocytopenia on hospital day 30. The patient is currently being managed with oral mycophenolate mofetil and hydroxychloroquine, and rehabilitation for higher brain dysfunction is planned. The course of the hospitalization is shown in the figure below (Figure 2).

## Discussion

SLE is a multisystem autoimmune disorder with a variable disease course and multiple clinical manifestations [4]. In this case, a diagnosis of acute encephalitis was made based on the patient’s symptoms, which included sudden seizures and impaired consciousness, and electroencephalography and brain MRI findings. Central nervous system infections were ruled out based on test results. Given the presentation of seizures in early adolescence and the presence of medial temporal lobe lesions, NMDARE was suspected. However, renal dysfunction and persistent thrombocytopenia persisted even after initiating treatment for NMDARE. Additional testing revealed positive ANA and low complement levels. It was concluded that the seizure and impaired consciousness at presentation manifested as neurological symptoms of SLE, not NMDARE.

SLE is a systemic disease that targets multiple organs and requires long-term treatment. When characteristic skin rashes are absent, as in this case, differential diagnosis based on physical findings alone is difficult. In this case, the only physical examination findings meeting the diagnostic criteria for SLE were fever and neurological symptoms. Upon further investigation, cytopenia, renal lesions, complement deficiency, and autoantibodies were identified. Understanding the diverse symptoms of SLE and not excluding it from consideration, even when only neurological symptoms are present are crucial.

The etiology of NP-SLE is thought to be a multifactorial condition involving the interaction of two pathways: direct neurotoxicity due to autoantibodies and cytokines, and microangiopathy (thrombosis and vasculitis) against the backdrop of blood-brain barrier (BBB) ​​breakdown. The bilateral symmetry of the MRI findings in this case strongly suggests systemic inflammation (autoimmunity) or metabolic factors rather than impaired blood flow. Given this case, one can consider the possible hypotheses/pathophysiology. (1) Small vessel disease: multiple microangiopathies due to antiphospholipid antibodies and complement activation occur in the deep white matter, including the brainstem. (2) Antibody-mediated neurotoxicity: anti-NR2 antibodies and others act on NMDA receptors in the hippocampus and insular cortex, thereby inducing status epilepticus owing to excitotoxicity. (3) Vasogenic edema: symmetric vasogenic edema occurs owing to widespread BBB breakdown. Thrombotic microangiopathy (TMA) is a complication of SLE and has been reported to develop concurrently with SLE disease activity [5].

Clinically, this is difficult to diagnose because NP-SLE and NMDARE both present with acute neurological symptoms, seizures, and impaired consciousness, making differentiation based on symptoms alone difficult. Furthermore, both diseases have a peak incidence in childhood and adolescence [6]. The treatment strategies for NP-SLE and NMDARE share a common foundation: aggressive immunosuppression to mitigate neuroinflammation. In both conditions, high-dose corticosteroids and cyclophosphamide or rituximab are utilized to target the underlying autoimmune process [7]. NMDARE is a synaptic autoimmune disorder where autoantibodies target NMDA receptors in the brain, leading to their removal from synapses [8]. A mouse-derived monoclonal anti-dsDNA antibody has been reported to cross-react with NMDA receptor subunits NR2A and NR2B [9]. Moreover, anti-NR2 antibodies in the CSF are reportedly significantly elevated in patients with NP-SLE [10]. Therefore, antibodies against NMDARE and NP-SLE overlap, making differentiation between the two challenging when based on clinical symptoms and imaging findings alone. Patients with NMDARE often require prolonged hospitalization but can achieve substantial recovery over months to years if treated early [11]. In contrast, the prognosis of NP-SLE is more closely tied to systemic disease activity and the cumulative damage of the central nervous system, often resulting in persistent cognitive impairment [12]. They are distinct entities characterized by different autoantibody profiles; anti-NR2 antibodies are prevalent in NP-SLE, whereas anti-GluN1 antibodies are the hallmark of anti-NMDAR encephalitis [6]. In SLE, complement levels decrease via immune complexes, whereas in NMDARE, complement levels hardly decrease at all [13]. Therefore, measuring complement levels may be useful for differentiating between these two diseases. In the differential diagnosis of acute psychosis in young females, the absence of ANA serves as a critical clinical marker. Given that ANA is present in more than 95% of patients with SLE [14], a negative ANA result significantly lowers the pre-test probability of NP-SLE. In such cases, the clinical suspicion should promptly shift toward primary autoimmune encephalitides, most notably NMDARE, which typically lacks systemic autoimmune markers [12]. This clinical note was limited in terms of objectively assessing the patient’s psychiatric symptoms.

Lupus was the main differential or was strongly suspected versus NMDARE because of "low platelet count, etc." In this case, acute-phase blood tests on the second day of hospitalization revealed low platelet count (33,000/µL) and elevated D-dimer (42.39 µg/mL). Furthermore, the haptoglobin test performed on the sixth day of hospitalization was below the sensitivity threshold, suggesting that microvascular formation was likely present in this case. The persistence of higher-level cognitive impairments, such as short-term memory deficits, even after hematologic recovery with treatment, may be attributable to this condition. Furthermore, although complement measurements continued after discharge in this case, C4 levels remained persistently low without recovery. Low C4 copy number variants have been reported to increase the risk of SLE onset [15], suggesting that the patient also had a high risk of developing SLE.

## Conclusions

We report a case initially treated for NMDARE but later diagnosed with NP-SLE. SLE is a systemic disease requiring long-term treatment. Early diagnosis and therapeutic intervention are essential for preventing sequelae. However, in the absence of characteristic rashes, differential diagnosis based on physical findings is difficult. Understanding the diverse symptoms of SLE and evaluating all organs throughout the body to include them in the differential diagnosis is extremely important. ANA and complement level measurements may help differentiate NP-SLE from autoimmune encephalitis, such as NMDARE, and should be included in a workup of a young female patient presenting with encephalitis symptoms.

## References

[1] Siegel CH, Sammaritano LR (2024). Systemic lupus erythematosus: a review. JAMA.

[2] Levy DM, Kamphuis S (2012). Systemic lupus erythematosus in children and adolescents. Pediatr Clin North Am.

[3] Fanouriakis A, Kostopoulou M, Alunno A (2019). 2019 update of the EULAR recommendations for the management of systemic lupus erythematosus. Ann Rheum Dis.

[4] Khormi AA, Hijazi FT (2023). SLE initially presenting with neuropsychiatric manifestations and seizure, case report. Immun Inflamm Dis.

[5] Fujimura Y, Matsumoto M (2010). Registry of 919 patients with thrombotic microangiopathies across Japan: database of Nara Medical University during 1998-2008. Intern Med.

[6] Hirohata S, Tanaka K (2019). Differential expression of antibodies to NMDA receptor in anti-NMDA receptor encephalitis and in neuropsychiatric systemic lupus erythematosus. Lupus Sci Med.

[7] Wu YY, Feng Y, Huang Y, Zhang JW (2016). Anti-N-methyl-D-aspartate receptor encephalitis in a patient with systemic lupus erythematosus. J Clin Neurol.

[8] Kayser MS, Dalmau J (2016). Anti-NMDA receptor encephalitis, autoimmunity, and psychosis. Schizophr Res.

[9] DeGiorgio LA, Konstantinov KN, Lee SC, Hardin JA, Volpe BT, Diamond B (2001). A subset of lupus anti-DNA antibodies cross-reacts with the NR2 glutamate receptor in systemic lupus erythematosus. Nat Med.

[10] Arinuma Y, Yanagida T, Hirohata S (2008). Association of cerebrospinal fluid anti-NR2 glutamate receptor antibodies with diffuse neuropsychiatric systemic lupus erythematosus. Arthritis Rheum.

[11] Titulaer MJ, McCracken L, Gabilondo I (2013). Treatment and prognostic factors for long-term outcome in patients with anti-NMDA receptor encephalitis: an observational cohort study. Lancet Neurol.

[12] Dalmau J, Lancaster E, Martinez-Hernandez E, Rosenfeld MR, Balice-Gordon R (2011). Clinical experience and laboratory investigations in patients with anti-NMDAR encephalitis. Lancet Neurol.

[13] Barry H, Byrne S, Barrett E, Murphy KC, Cotter DR (2015). Anti-N-methyl-d-aspartate receptor encephalitis: review of clinical presentation, diagnosis and treatment. BJPsych Bull.

[14] Aringer M, Costenbader K, Daikh D (2019). 2019 European League Against Rheumatism/American College of Rheumatology classification criteria for systemic lupus erythematosus. Ann Rheum Dis.

[15] Mariaselvam CM, Seth G, Kavadichanda C (2024). Low C4A copy numbers and higher HERV gene insertion contributes to increased risk of SLE, with absence of association with disease phenotype and disease activity. Immunol Res.

